# North of England Medical and Surgical Journal

**Published:** 1830-04-01

**Authors:** 


### LXII.

### NORTH OF ENGLAND MEDICAL AND SURGICAL JOURNAL.

We have just received the prospectus of this new contemporary, which is to be in the quarterly form, and to consist almost entirely of original communications. The address is written in an excellent tone of feeling. The journal is to be "exclusively devoted to science, to the total exclusion of *party politics and personal allusions* and it will be its constant aim to cultivate that *high tone of moral feeling and mutual respect*, which should ever distinguish the members of a liberal profession." This looks well; and we do hope that the members of our profession in the great northern counties will cordially support by their contributions, a work whose objects are so dignified and useful. The first num berwill appear this Spring, and four numbers (price 2s. 6d. each) will constitute a handsome octavo volume. We observe that communications are to be addressed to Mr. Sowler, bookseller, Manchester, or Mr. Knight, bookseller, Leeds. We shall be happy to give our new contemporary every facility and assistance in our power.

